# Non-Thermal Hydrodynamic Cavitation for Surplus Fruits and Vegetables: Improved Vitamin C and Bioactive Preservation

**DOI:** 10.3390/foods15020268

**Published:** 2026-01-12

**Authors:** Julian Quintero-Quiroz, Natalia Zuluaga-Arroyave, Alejandra Valencia-Naranajo, María C. Molina-Castillo, Nathalia Varela-Garcia, Mauricio Medina-Rodriguez, Jaison Martínez-Saldarriaga, Juan Camilo Henao-Rojas

**Affiliations:** 1 College of Food and Pharmaceutical Sciences, CES University, Calle 10 # 22-04, Medellin 050018, Colombia; nzuluaga@ces.edu.co (N.Z.-A.); avalencian@ces.edu.co (A.V.-N.); molina.maria@uces.edu.co (M.C.M.-C.); varela.nathalia@uces.edu.co (N.V.-G.); medina.mauricior@uces.edu.co (M.M.-R.); 2 Corporación Colombiana de Investigación Agropecuaria (AGROSAVIA)—C.I. La Selva, Km 7 vía Las Palmas, Rionegro 054048, Colombia; jmartinezs@agrosavia.co; 3 Grupo de Investigación en Sustancias Bioactivas, Facultad de Ciencias Farmacéuticas y Alimentarias, Universidad de Antioquia UdeA, Calle 70 No. 52-21, Medellin 050010, Colombia

**Keywords:** hydrodynamic cavitation, functional foods, agri-food surplus valorization

## Abstract

This study evaluated the impact of hydrodynamic cavitation (HC) versus conventional thermal processing (TT) for the valorization of fruit and vegetable surpluses, using optimized purees of carrot, banana, yacón, beetroot, and gulupa. HC-treated purees consistently preserved bioactive compounds, with vitamin C retention in purple carrot puree reaching 6.8 ± 0.6 mg/100 g, compared to only 0.6 ± 0.0 mg/100 g after thermal treatment. Total polyphenol content and antioxidant capacity (FRAP up to 2580 ± 126 μmol Eq-Trolox/100 g, DPPH inhibition up to 88.72% ± 0.80) were similarly superior with HC. While HC resulted in noticeably higher grumosity and fibrosity, limiting acceptance, TT improved sensory sweetness but degraded nutritional quality, causing up to 80% losses of vitamin C and bioactives. The findings confirm that HC is an effective non-thermal strategy for converting agri-food surpluses into functional ingredient bases, maximizing nutritional retention and energetic efficiency and supporting sustainable circular food systems.

## 1. Introduction

Food loss and waste (FLW) is a major threat to the sustainability of the global food system, arising from losses along production–processing chains and waste at retail and household levels. Globally, around one-third of food produced for human consumption, about 1.3 billion tonnes per year is lost or wasted, generating roughly 1.05 billion tonnes of waste (≈19% of available food) and 8–10% of global greenhouse gas emissions. In Colombia, FLW reaches 9.76 million tonnes annually (34% of national production), 62% of which corresponds to fruits and vegetables, while about 26% of households experience food insecurity [1]. Valorizing fruit and vegetable surpluses through innovative processing can better exploit their content of bioactive compounds, dietary fiber, and essential minerals than conventional uses such as composting or animal feed.

The effectiveness of these strategies depends critically on the processing technology. Conventional thermal operations (e.g., pasteurization 75–95 °C, sterilization 110–115 °C) ensure safety and shelf life but cause severe degradation of thermolabile nutrients (vitamin C losses up to 50–70%, B vitamins 20–40%), destruction of phenolics, anthocyanins, and carotenoids, undesirable sensory changes, and high energy demand, particularly problematic for fruit–vegetable matrices where fresh-like quality is desired. Hydrodynamic cavitation (HC) has emerged as a non-thermal or minimally thermal alternative capable of microbial inactivation, enzyme deactivation, and structural modification while better preserving nutritional and functional attributes [2]. HC generates microbubbles by pressure drops in a flowing liquid; their subsequent collapse produces localized extreme conditions (high temperature and pressure, shock waves, microjets, and reactive oxygen species) that promote turbulence, shear, cell disruption, and controlled radical formation without substantially increasing bulk temperature, thereby limiting thermal degradation of sensitive bioactives [3,4].

Evidence from fruit-based systems shows that HC can enhance physicochemical stability and preserve bioactive compounds. In apple-derived products, HC has yielded improved homogenization, lower viscosity, and greater stability than thermal treatments, with higher antioxidant activity after refrigerated storage, and has increased the release and bioaccessibility of polyphenols compared with conventional heating. In other juices and plant-based beverages, HC has achieved 2–6 log reductions in vegetative pathogens at temperatures 20–30 °C below those of conventional processes, while maintaining or improving carotenoids, hesperidin, phenolics, and overall antioxidant capacity relative to pasteurization. These benefits are attributed to the combination of mild thermal impact and disruption of plant tissues, which facilitates the release of bound bioactives and can reduce energy use [5,6,7,8,9].

Despite this progress, knowledge gaps remain. Most studies focus on single commodities rather than complex multicomponent matrices that better represent real surplus streams, and conventional assays such as total phenolics and bulk antioxidant capacity provide only a partial picture of HC-induced biochemical changes. Metabolomic profiling using advanced chromatographic and mass spectrometric techniques offers a more detailed view of low-molecular-weight metabolites, enabling the assessment of both degradation and enhanced release of target compounds under HC processing [10,11].

Building on this context, the present work investigates a multicomponent matrix comprising purple carrot (*Daucus carota* var. *atrorubens*), orange carrot (*Daucus carota*), banana (*Musa* spp.), yacón (*Smallanthus sonchifolius*), gulupa (*Passiflora edulis*), and beetroot (*Beta vulgaris*), representative of surpluses recovered in Colombia through REAGRO–Colombia Food Bank programs and Agrosavia materials. The main objective is to evaluate the effect of hydrodynamic cavitation, compared with conventional thermal treatment, on the physicochemical and bromatological properties, antioxidant capacity, metabolomic profile, and sensory attributes of a mixed fruit–vegetable purée, thereby assessing HC as a tool to convert agri-industrial surpluses into high-quality functional ingredients that support circular economy and food security.

## 2. Materials and Methods

### 2.1. Samples and Chemicals

All fruit and vegetable samples used in this study were sourced as surplus material from regional suppliers and the Colombia Food Bank and Agrosavia. Purple carrot (*Daucus carota* var. *atrorubens*), orange carrot (*Daucus carota* L.), banana (*Musa* spp.), yacón (*Smallanthus sonchifolius*), beetroot (*Beta vulgaris*), and gulupa (*Passiflora edulis*) were washed thoroughly with potable water and stored at 4 ± 2 °C pending use. Each material was diced into uniform pieces prior to thermal or hydrodynamic treatment to ensure homogeneity across experimental runs. All reagents used for physicochemical and antioxidant analyses were of analytical grade and sourced from Sigma-Aldrich (St. Louis, MO, USA) and Merck (Darmstadt, Germany). These included Folin–Ciocalteu reagent, gallic acid, 2,2-diphenyl-1-picrylhydrazyl (DPPH), Trolox, sodium carbonate, 2,2′-azobis (2-amidinopropane) dihydrochloride (AAPH), fluorescein, ascorbic acid, acetone, petroleum ether, and standard solutions for mineral and vitamin determinations. Distilled water for solution preparation and assays was purified by reverse osmosis.

### 2.2. Experimental Fruit and Vegetable Mixed Design

The effect of hydrodynamic cavitation on the physicochemical and functional properties of mixed fruit and vegetable purées was optimized using a Simplex Lattice design with a quadratic model in Design-Expert 12 (Stat-Ease Inc., Minneapolis, MN, USA). Two independent designs were implemented: one for purple carrot-based purée (*Daucus carota* var. *atrorubens*) and another for orange carrot-based purée (*Daucus carota* L.). The purées comprised five cavitated components: carrot (Component A; purple or orange), banana (B), yacón (C), beetroot (D), and gulupa (E). Component proportions ranged from 0 to 1.0 in 0.25 increments (0–100% *w*/*w*), generating 31 randomized experimental runs per design (Table 1). For each run, the required masses of each cavitated component were weighed, combined, and homogenized according to the design.

Five response variables were evaluated: color (colorimeter), antioxidant capacity by DPPH (% inhibition) and FRAP (µmol Eq-Trolox/100 g), total polyphenolic content (TPC, mg GAE/g, Folin–Ciocalteu method), and apparent viscosity (cP, viscometer). Multiple linear regression (least squares) was used to model responses; model adequacy was assessed by r^2^ and adjusted r^2^, and term significance by ANOVA at 95% confidence. Optimization targeted maximum antioxidant indicators (DPPH, TPC, FRAP) and L* values compatible with projected consumer acceptance. The methodology used in the response variables and Hydrodynamic cavitation treatment are described below.

#### 2.2.1. Hydrodynamic Cavitation Treatment

**Figure 1:**
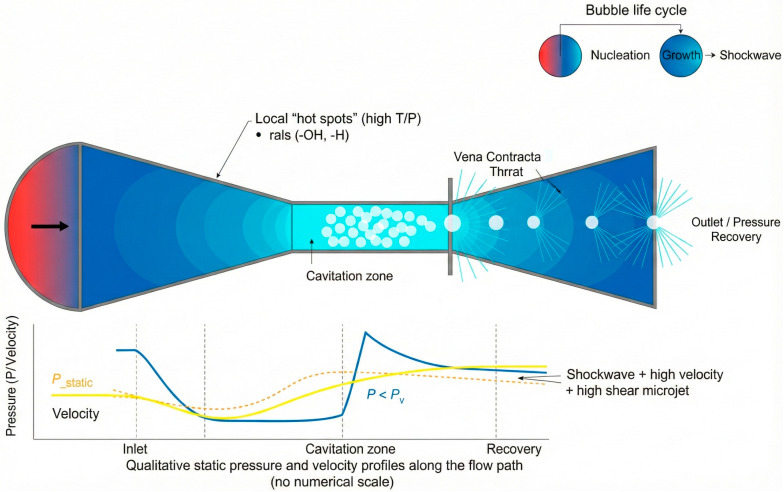
Schematic representation of the cavitation mechanism in food systems, illustrating the key phenomena of bubble formation, the thermodynamic cycle, and the subsequent mechanical effects.

Plant-based matrices were introduced into the cavitation system following a pre-milling step using a rotor drum mill equipped with stainless steel blades (3400 rpm; Creativa Ingeniería, Medellin, Colombia). This milling unit was directly coupled to the hydrodynamic cavitation reactor model TEK 1 SM-SS-SD (TeckMash-KAVITEC, Kharkiv, Ukraine), which has a maximum processing capacity of 32.5 L. Operational parameters for cavitation were standardized prior to processing. The system was configured to operate at a maximum temperature of 85 °C, a working pressure of 5 PSI, and a residence time of 5 min. The total processing cycle lasted 16 min, accounting for stabilization and product discharge phases. All functional ingredients and additives were incorporated directly into the receiving tank prior to cavitation, based on optimal formulation parameters established in prior experimental design studies. The system was then hermetically sealed to ensure consistent hydrodynamic conditions. Upon completion of the cavitation cycle, the processed material was evacuated from the HTD reactor and transferred immediately to a downstream packaging system to preserve the functional and sensory integrity of the final product (Figure 1). 

#### 2.2.2. Colorimetry Analysis

Color attributes (L*, a*, b*, C*, h°) of the final products were measured in triplicate using a ColorFlex EZ spectrophotometer (Hunter Associates Laboratory, Reston, VA, USA), calibrated with standard black and white tiles before each use. Results were recorded in both CIELab systems. CIE Lab* system, where L* (0–100) represents lightness, a* the green (−)/red (+) axis, b* the blue (−)/yellow (+) axis, C* chroma $\left(C^{*}=(a^{*2}+b^{*2}{)}^{1/2}\right)$, and h° the hue angle in degrees $\left(h^{\circ }=arctan(b^{*}/a^{*})\right)$.

#### 2.2.3. Total Phenolic Content (TPC)

TPC was determined using the Folin–Ciocalteu colorimetric method, following minor adaptations as described by Zapata-Vahos et al. (2023) [12]. Briefly, 50 µL of sample were mixed with 425 µL distilled water and 125 µL Folin–Ciocalteu reagent, left for 6 min, followed by 400 µL of 7.1% (*w*/*v*) Na_2_CO_3_. After 1 h at room temperature in the dark, absorbance was read at 760 nm. Quantification was performed with a gallic acid calibration curve; results were expressed as mg GAE/100 g sample [12].

#### 2.2.4. DPPH Radical Scavenging Activity

DPPH radical scavenging capacity was measured as described by Brand-Williams et al., with adaptations from Zapata-Vahos et al. (2023) [12]. Briefly, 10 µL sample was mixed with 990 µL DPPH solution, reacted for 30 min at room temperature, and absorbance measured at 517 nm. Radical inhibition (%) was calculated vs. reference, and antioxidant activity was reported as µmol Trolox equivalents (TEAC) per 100 g sample [12].

#### 2.2.5. Ferric Reducing Antioxidant Power (FRAP)

FRAP was evaluated according to Benzie and Strain, with modifications from Zapata-Vahos et al. (2023) [12]. In brief, 50 µL sample were combined with 950 µL FRAP reagent, incubated 30 min at room temperature, and absorbance determined at 593 nm. Quantification was performed using an ascorbic acid calibration curve, and results were reported as mg ascorbic acid equivalents (AEAC) per 100 g sample [12].

#### 2.2.6. Apparent Viscosity by Viscometer

Apparent viscosity was determined in triplicate at room temperature using a Brookfield Digital Laboratory Viscometer model DH-DJ-8S (Brookfield, Middleboro, MA, USA). Samples were placed in the equipment’s vessel, and the spindle was selected based on achieving approximately 50% torque, in accordance with the manufacturer’s recommendations. Measurements were conducted at a constant speed, and results were expressed in centipoise (cP).

### 2.3. Characterization of Purees by Cavitation and Thermal Analysis

After the optimal formulation was defined by experimental design, two types of fruit and vegetable purees were produced; one by hydrodynamic cavitation and the other by thermal treatment. For the thermal treatment, all fruit ingredients (including whole banana, carrot, and gulupa with peel) were blanched and then subjected to heat at 90 °C for 90 min until they were sufficiently softened. The softened fruits were then homogenized using a Thermomix (Vorwerk, Wuppertal, Germany) for 10 min at controlled temperature. The cavitated puree was prepared by subjecting the mixed fruit and vegetable according to the determined optimal formulation, to hydrodynamic cavitation, as previously described. Both purees were cooled and stored until analysis.

Both resulting products were evaluated by the full suite of physicochemical, nutritional, rheological, and functional property tests such as of pH, Brix, moisture, ash, protein, fat, total carbohydrates, dietary fiber, vitamin C, minerals (Fe, Ca, Zn), titratable acidity, texture profile (firmness, consistency, cohesiveness, cohesion work), color coordinates (L*, a*, b*, C*, h°), rheological properties (apparent viscosity, flow index, consistency coefficient, yield stress, r^2^), antioxidant capacity (FRAP, DPPH % inhibition, TPC, ORAC, beta-carotene), and sensorial assessment. All determinations were conducted in triplicate, as described in the detailed protocols for each parameter. The missing methodologies are described below.

#### 2.3.1. Analysis of Physical-Chemical Properties

The pH, °Brix, moisture, ash, protein, fat, total carbohydrates, dietary fiber, vitamin C, minerals (Fe, Ca, Zn), and titratable acidity of the purées were determined using standard AOAC methods. Moisture was measured gravimetrically at 105 °C to constant weight (AOAC 934.06 [13]); protein by the Kjeldahl method (AOAC 2011.04 [14]); ash by incineration at 550 °C (AOAC 942.05 [15]); and fat by Soxhlet extraction (AOAC 922.06 [16]). Total carbohydrates were calculated by difference, dietary fiber was quantified enzymatically (AOAC 985.29 [17]), and vitamin C and minerals were analyzed using the corresponding AOAC procedures. Titratable acidity was determined by titration with standardized NaOH. All analyses were performed in triplicate [12].

#### 2.3.2. Determination of Apparent Viscosity by Rheometer

The rheological properties were analyzed using an Anton Paar MCR 92 rheometer (Anton Paar GmbH, Graz, Austria) equipped with Rheocompass^®^ software (v1.20, Anton Paar, Graz, Austria) and a C-CC27 concentric cylinder geometry (27 mm diameter). Approximately 20 mL of each sample were loaded, and flow curves were recorded at 25 °C using three phases: an ascending shear rate from 0.01 to 100 s^−1^ for 60 s, a holding period at 100 s^−1^ for 60 s, and a descending rate from 100 to 0.01 s^−1^ for 60 s. Data from the descending curve were fitted to the Herschel–Bulkley model to obtain the consistency index (K), flow behavior index (n), and apparent viscosity at 50 s^−1^. All measurements were performed in triplicate [18].

#### 2.3.3. Texture Profile Analysis

Textural properties were measured at ambient temperature using a TA.XT Plus texture analyzer (Stable Micro Systems Ltd., Surrey, UK) with a 35 mm back-extrusion ring. The assay quantified firmness (Firm), consistency (Con), cohesiveness (Coh), and viscosity index (Wcoh) as key mechanical descriptors of sample behavior under deformation, using a trigger force of 5 g, crosshead speeds of 1, 5, and 10 mm s^−1^, and 20 mm probe displacement, according to Sert et al. (2017) [19]. Results are the mean of at least three replicates [19].

#### 2.3.4. Determination of Antioxidant Activity by ORAC Assay

Oxygen radical absorbance capacity (ORAC) was measured to assess antioxidant activity. In 96-well microplates, 80 µL of sample, 80 µL of fluorescein solution (14.66 ppm), and 40 µL of AAPH (120 mg/mL) were mixed. The reaction was incubated at 37 °C in a Victor^®^ 1420 Multilabel Plate Counter (PerkinElmer, Waltham, MA, USA), recording fluorescence (λex = 485 nm, λem = 535 nm) every 5 min over 180 min. Antioxidant capacity was calculated using a standard curve prepared with 20 µM Trolox. All measurements were performed in triplicate [20].

#### 2.3.5. Determination of Beta-Carotene Content

Beta-carotene content was determined by spectrophotometry following organic solvent extraction. Briefly, an aliquot of sample was extracted with acetone/petroleum ether, and the upper (organic) phase was collected. The absorbance of the extract was measured at 450 nm in 1 cm path length quartz cuvettes using a UV-Vis spectrophotometer (UV-1700, Shimadzu Corporation, Kyoto, Japan). Beta-carotene concentration was calculated from a calibration curve prepared with pure beta-carotene standards under identical conditions. Results were expressed as mg beta-carotene per liter of extract. All measurements were performed in triplicate [21].

#### 2.3.6. Sensory Profile Evaluation

The sensory profile evaluation was conducted following the general guidelines of ISO 13299:2016 [22]. A panel of 20 trained assessors evaluated all samples using structured scales with reference levels: 0 to 10 points for sensory attributes and 0 to 5 points for overall acceptance, both calibrated against sensory references. Samples were presented in randomized, coded order at ambient temperature in standardized white cups. Each panelist evaluated samples in triplicate with appropriate palate-cleansing intervals between tastings. Since the variables did not follow a normal distribution, scores were reported as medians (Me) and interquartile ranges (IQR). Differences in attribute medians were analyzed with Fisher’s exact test, using a statistical significance threshold of *p* < 0.05.

### 2.4. Targeted Metabolomic Profiling of Phenolic Compounds by LC–QqQ–MS

A targeted metabolomic analysis of phenolic compounds was carried out on the carrot-based purées processed either by hydrodynamic cavitation (HC) or conventional thermal treatment (TT). For each formulation and processing technology, three independent batches were produced, and analytical determinations were performed at least in triplicate.

#### 2.4.1. Sample Preparation

For metabolite extraction, 100 mg of each freeze-dried purée were accurately weighed into 2 mL microtubes and mixed with 500 µL of a methanol–water solution (80:20, *v*/*v*) containing 0.1% formic acid. The suspensions were vortex-mixed for 10 min at 3200 rpm to ensure complete dispersion of the matrix, followed by sonication for 10 min in an ultrasonic bath to enhance the extraction of phenolic compounds. The resulting extracts were centrifuged, and the supernatants were filtered through 0.22 µm PTFE syringe filters into amber LC vials for immediate analysis by LC–MS.

#### 2.4.2. LC–QqQ–MS Conditions

Chromatographic separation was performed on an Agilent 1260 Infinity LC system coupled to a 6470 triple-quadrupole mass spectrometer with an electrospray ionization source (Agilent Technologies Inc., Santa Clara, CA, USA). An InfinityLab Poroshell 120 EC-C18 column (2.1 × 150 mm, 2.7 µm; Agilent Technologies Inc., Santa Clara, CA, USA) was operated at 30 °C. The mobile phase consisted of water with 0.1% formic acid (A) and acetonitrile with 0.1% formic acid (B), at 0.4 mL/min, using a gradient from 2% to 30% B (0–10 min), then to 98% B (10–20 min, held 2 min), followed by re-equilibration for 5 min; injection volume was 3 µL. The mass spectrometer acquired data in multiple reaction monitoring (MRM) mode under positive and negative ESI to target phenolic acids, flavonoids, and related compounds. Source parameters were optimized for sensitivity while maintaining chromatographic resolution. Analytical standards (purity ≥ 97%) were obtained from MetaSci (Toronto, ON, Canada); compound-specific MRM transitions, collision energies, and retention times are listed in Supplementary Table S3.

#### 2.4.3. Metabolomic Data Processing

Chromatographic data were processed with MassHunter software (version 10.0, Agilent Technologies Inc., Santa Clara, CA, USA), with automatic peak integration followed by manual inspection when required. Peak areas for each targeted metabolite were normalized to sample dry weight and semi-quantified using external calibration curves prepared from serial dilutions of the corresponding authentic standards. Results were expressed as µg equivalent of each standard per g of dry sample. The normalized metabolite profiles were then used for univariate and multivariate analyses (PLS-DA) to compare HC- and TT-processed purées and to identify phenolic compounds most affected by processing.

### 2.5. Statistical Analysis of Processing Technologies: Traditional vs. Hydrodynamic Cavitation

All quantitative response variables were first tested for normality (Shapiro–Wilk, α = 0.05) and homoscedasticity (Levene, α = 0.05) to verify the assumptions for parametric analyses. Subsequently, one-way ANOVAs were performed separately for orange and purple carrot to evaluate the effect of processing technology (Traditional vs. Hydrodynamic Cavitation) on each response variable, using a completely randomized design. When significant differences were detected (*p* < 0.05), Tukey’s HSD post hoc test (α = 0.05) was applied. Results were visualized as bar plots with standard error bars and grouping letters.

In parallel, Partial Least Squares Discriminant Analysis (PLS-DA) was used to explore the joint contribution of all variables to sample classification by processing technology, with two latent components per plant matrix. Model performance was described by R^2^X, R^2^Y, and Q^2^ (leave-one-out cross-validation). Variable Importance in Projection (VIP) scores were calculated, and variables with VIP > 1.0 were considered most discriminant. All analyses and graphics were generated in Python 3.10 using scientific libraries.

## 3. Results

### 3.1. Experimental Design and Sample Processing

**Figure 2:**
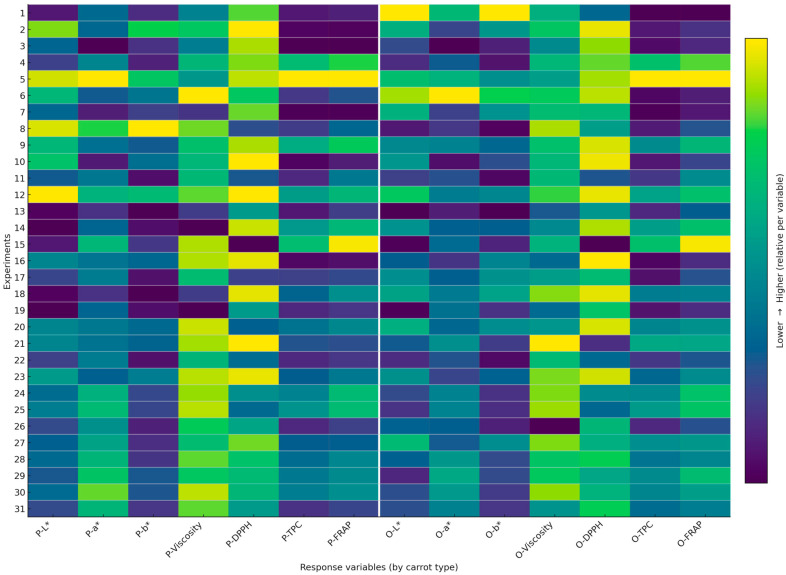
Heatmap of measured response variables across the experimental matrix for purple (P-) and orange (O-)carrot purée formulations. Viscosity: expressed in cP, DPPH expressed in Inhibition (%), TPC expressed in mg EAG/g, FRAP expressed in µmol Eq-Trolox/100 g.

A total of 31 experimental runs were performed for both the purple and orange carrot purée formulations, as defined by the Simplex Lattice mixture design. All experiments were conducted in triplicate, and Figure 1 summarizes the mean response patterns across runs using a heatmap representation. For both formulations, the table summarizes the response variables, including luminosity (L*), a*, b*, viscosity, DPPH (% inhibition), total polyphenolic content expressed in mg EAG/g (TPC), and FRAP values expressed in µmol Eq-Trolox/100 g. To facilitate visual comparison, the color intensity in Figure 2 reflects relative (within-variable) variation across experiments rather than absolute values. The complete table with mean values is provided in the Supplementary Material (Table S1). These data reflect the broad experimental space explored for both carrot types and allow precise comparison across mixture compositions.

For the purple carrot puree formulation, the highest antioxidant capacity as measured by DPPH inhibition was 88.72% (±0.80), while the highest FRAP value was 887.11 µmol Eq-Trolox/100 g (±2.95). Both maxima were observed in different mixture runs, highlighting the impact of component variations on antioxidant performance. In the orange carrot puree, the maximum DPPH inhibition reached 88.72% (±0.80) and the highest FRAP was also 887.11 µmol Eq-Trolox/100 g (±2.95), occurring in analogous mixture runs to the purple carrot case. These results demonstrate that both carrot bases, when optimized, can deliver high antioxidant activities under suitable component combinations.

Color and viscosity in both purple and orange carrot purees were strongly influenced by the ratios of fruit and vegetable components within each formulation. The luminosity (L*) was highest in blends with a greater proportion of gulupa, especially in the purple carrot system (L* = 34.51 ± 0.01), enhancing the brightness and stability of color. The orange carrot purees showed a similar but generally lighter hue, reflecting the higher carotenoid and lower anthocyanin content compared to purple carrot. Viscosity reached maximum values (up to 39,330 cP ± 2163.75) in formulations dominated by carrot, while increasing the share of banana or yacon led to lower viscosity, favoring improved texture and processing, consistent with their higher soluble solids and lower fiber fractions. Thus, optimal combinations balanced vivid color, stable brightness, and manageable viscosity, key for product functionality and sensory quality.

The ANOVA results (Table 1) confirmed that the mixture models for all target variables in both purple and orange carrot purees were highly significant, with overall model *p*-values below 0.0001 for every property assessed. For purple carrot, coefficients of determination (R^2^ = 0.95–0.98) and adjusted R^2^ (0.89–0.92) confirmed robust fit across all responses.

Among mixtures, the AD interaction was particularly relevant for L* and DPPH inhibition (*p*-values = 0.0098 and 0.0001, respectively), while CD significantly affected b* (*p*-values = 0.029) and TPC (*p*-values = 0.0429). The DE interaction was strongly associated with DPPH (*p*-values = 0.0001) and FRAP (*p*-values < 0.0001), highlighting the combined influence of purple carrot with beetroot or gulupa on boosting both color and antioxidant properties. Positive main effects from carrot components were further enhanced by the addition of these fruits, as evident in multiple significant model terms. In the orange carrot system, global model fits remained highly significant (model *p*-values < 0.0001; R^2^ = 0.88–0.97; adjusted R^2^ = 0.83–0.96). Significant effects included main mixture terms such as AB (L*, *p*-values = 0.0019; TPC, *p*-values = 0.0292), AD (a*, *p*-values = 0.0089; DPPH, *p*-values = 0.0292), and AE (b*, *p*-values = 0.0078), indicating that blends with banana, beetroot, or yacon were key drivers of color and total polyphenol content.

**Figure 3:**
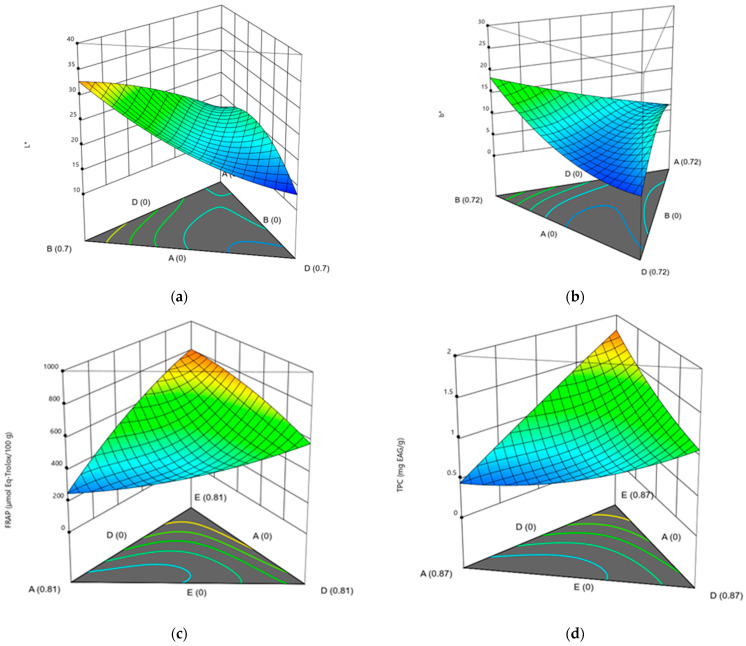

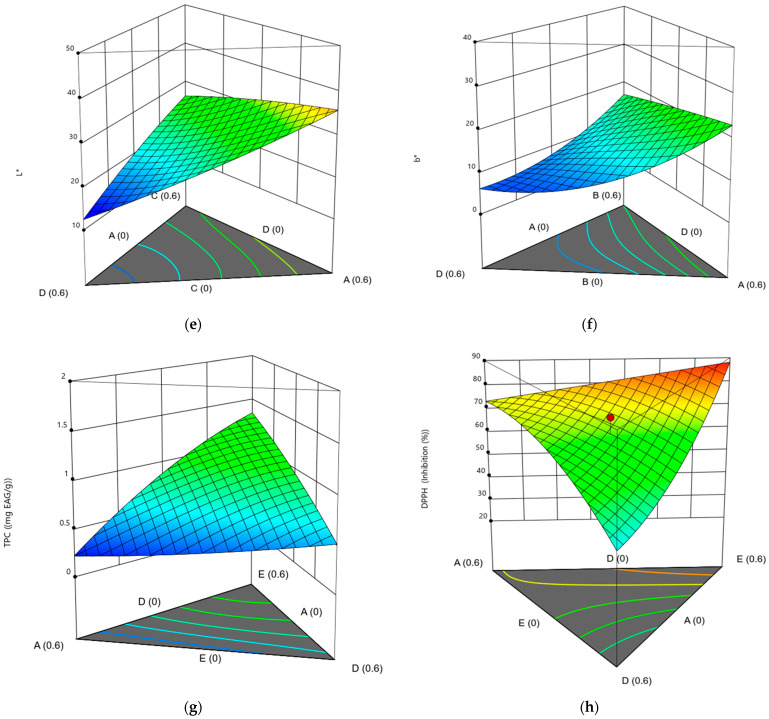
Response surface plots for key technological and functional variables as a function of mixture composition: (**a**) Luminosity (L*) for purple carrot puree; (**b**) Luminosity (L*) for orange carrot puree; (**c**) FRAP value (µmol Eq-Trolox/100 g) for purple carrot puree; (**d**) Total polyphenolic content (TPC, mg EAG/g) for purple carrot puree; (**e**) Luminosity (L*) for orange carrot puree. (**f**) FRAP value (µmol Eq-Trolox/100 g) for orange carrot puree; (**g**) Total polyphenolic content (TPC, mg EAG/g) for orange carrot puree; and (**h**) DPPH inhibition (%) for orange carrot puree.

The CD interaction was also significant for FRAP (*p*-values = 0.0125), emphasizing the contribution of beetroot in maximizing antioxidant capacity when paired with orange carrot. Collectively, the statistical evidence demonstrated that maximizing antioxidant and color properties in carrot-based purees requires carefully balancing carrot with high-activity partners (gulupa, beetroot, banana, yacon) to harness favorable main and synergistic effects, as visualized in the response surface plots (Figure 3).

The optimization of the experimental designs for both purple and orange carrot puree formulations was achieved with high predictive accuracy, as indicated by absolute errors exceeding 86% for all primary response variables. The optimal mixtures identified for the purple carrot compote consisted of 46% carrot, 40% banana, 7% yacon, 3% beetroot, and 4% gulupa, resulting in balanced color (L* = 24.57), notable antioxidant activity (DPPH = 76.98%, FRAP = 281.54), and moderate viscosity (44,494 cP). For the orange carrot system, the optimal blend was 51% carrot, 35% banana, 5% yacon, 4% beetroot, and 5% gulupa, yielding a brighter color (L* = 36.26), strong antioxidant capacity (DPPH = 75.03%, FRAP = 189.13), and lower viscosity (34,076 cP). These optimized formulations represent robust targets for formulation, as they were selected under criteria that achieved maximal desirability (D = 1.00), while maintaining all compositional and functional variables within defined acceptability limits.

### 3.2. Characterization of Purees by Cavitation and Thermal Analysis

**Figure 4:**
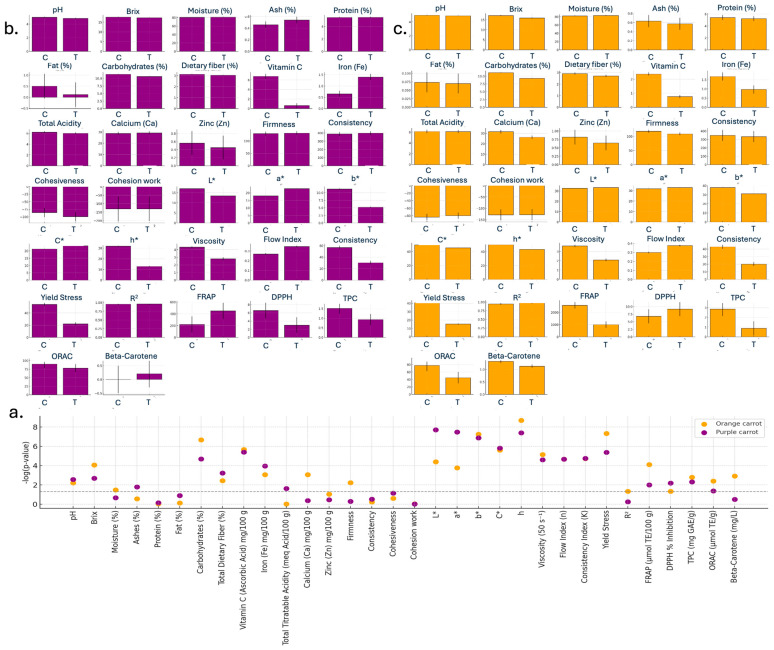
Statistical plots for physicochemical, color, texture, and bioactive compounds in carrot purées (cavitation vs. thermal). (**a**) −log_10_(*p*-value) significance plot comparing hydrodynamic cavitation (C) and thermal treatment (T) across variables. (**b**) Small-multiple bar plots showing mean values (±variability) for each variable under C and T (purple carrot); Supplementary Table S2. (**c**) Small-multiple bar plots showing mean values (±variability) for each variable under C and T (orange carrot); Supplementary Table S2.

A comprehensive physicochemical, colorimetric, texture, and bioactive compound characterization was performed on both purple and orange carrot purées subjected to hydrodynamic cavitation and conventional thermal treatments. Figure 4 and the Supporting Information Table S1 provide a summary of all measured parameters, including pH, °Brix, moisture, ash, protein, fat, carbohydrates, dietary fiber, vitamin C, minerals (Fe, Ca, Zn), titratable acidity, color coordinates (L*, a*, b*, C*, h°), texture properties (firmness, consistency, cohesiveness, cohesion work), rheological parameters (apparent viscosity, flow index, consistency coefficient, yield stress), and the principal indicators of antioxidant capacity (DPPH inhibition, FRAP, TPC, β-carotene).

Hydrodynamic cavitation treatment preserved a significantly higher content of vitamin C and polyphenols, including greater antioxidant activity (ORAC) in both matrices compared to thermal processing. In terms of vitamin C content, purple puree reported a preservation of 973.02% and orange puree 212.15%, related to a higher amount of this nutrient present in cavitated formulations. TPC reported a similar result, whereas cavitated orange puree reported a great preservation percentage (227.20%) compared to thermically treated; however, cavitated purple puree presented a minor preservation percentage close to 62.61% in a similar comparison to the thermically treated purple one. Antioxidant capacity associated with ORAC analysis also resulted in greater antioxidant activity for both cavitated purees, reaching preservation percentages of 73.90% and 15.22% in orange and purple samples, respectively. In addition, the orange carrot puree achieved a mean FRAP value of 2580 ± 126 μmol Eq-Trolox/100 g versus 993 ± 110 μmol Eq-Trolox/100 g in the thermally treated counterpart (*p*-values < 0.001), representing a 159.68% preservation under hydrodynamic cavitation treatment. DPPH inhibition followed a similar trend for purple puree, confirming superior retention of antioxidant potential by non-thermal technology, representing a 118.14% antioxidant capability preservation.

Color hue (h°) was significantly higher in cavitated purple purée (31.90 ± 0.09°) than in thermally treated purée (12.87 ± 0.28°), indicating a shift from a deep purplish-red towards a more reddish–yellow hue rather than a direct preservation of the original purple color. In orange purée, differences in h° between treatments were comparatively smaller (15.31% variation), suggesting a more limited impact of processing on hue. Brightness (L*) showed similar values in orange carrot purées (32.6–33.4), whereas in purple formulations cavitation resulted in lower L* (17.09 ± 0.05) than thermal treatment (13.49 ± 0.01), indicating a darker and visually more intensely colored product [23]. In terms of texture, both the treatment and carrot material purees consistently exhibited higher firmness (up to 132 ± 1 N) and consistency (up to 300 ± 1 N), contributing to desirable rheological behaviors observed in apparent viscosity and flow in Figure 4; however, viscosity, yield stress, and consistency index reported greater values in cavitated treatment for orange and purple purees. Apparent viscosity presented an increase of 71.65% and 54.45% for orange and purple puree, respectively. In a similar way, the consistency index reported increased values in both carrot samples, 133.16% for the orange sample, and 87.44% for the purple sample. Finally, yield stress remarkably increases percentages, reporting 167.68% for orange puree and 144.30% for purple puree.

**Figure 5:**
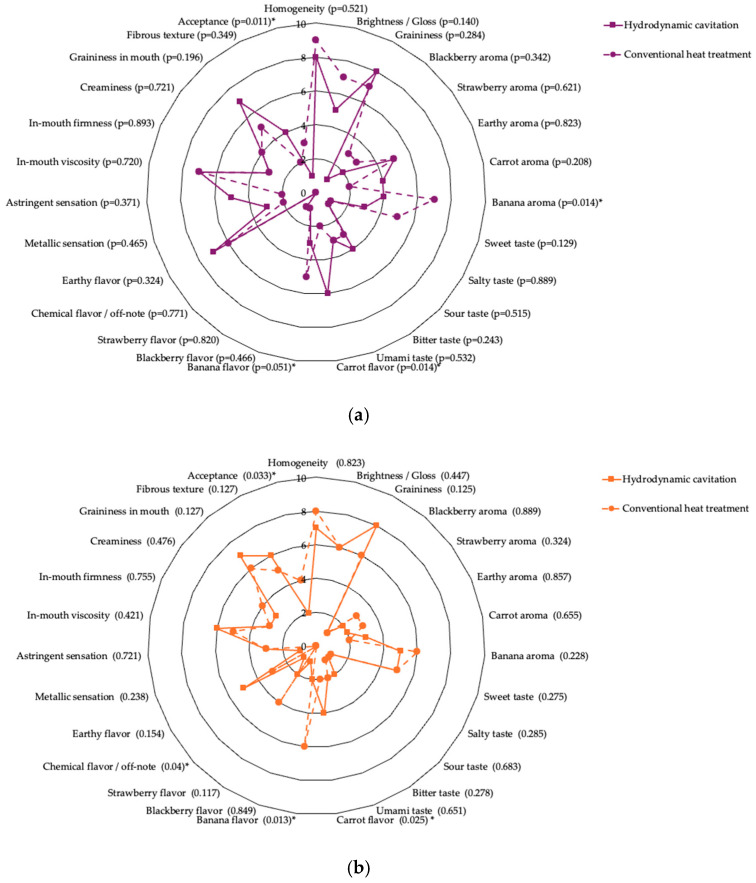
Sensory analysis boxplots (medians, IQRs) for carrot puree products. (**a**). Purple carrot, cavitation/traditional, (**b**). Orange carrot, cavitation/traditional; * represents a statistically significant difference with a *p*-values < 0.05.

The sensory analysis (Figure 5) evidenced higher median scores for flavor, texture, and acceptability in cavitated products, both for purple and orange carrot bases. Notably, overall acceptance was significantly greater in the hydrodynamically processed purees, as reflected by Fisher’s test (*p*-values < 0.05).

**Figure 6:**
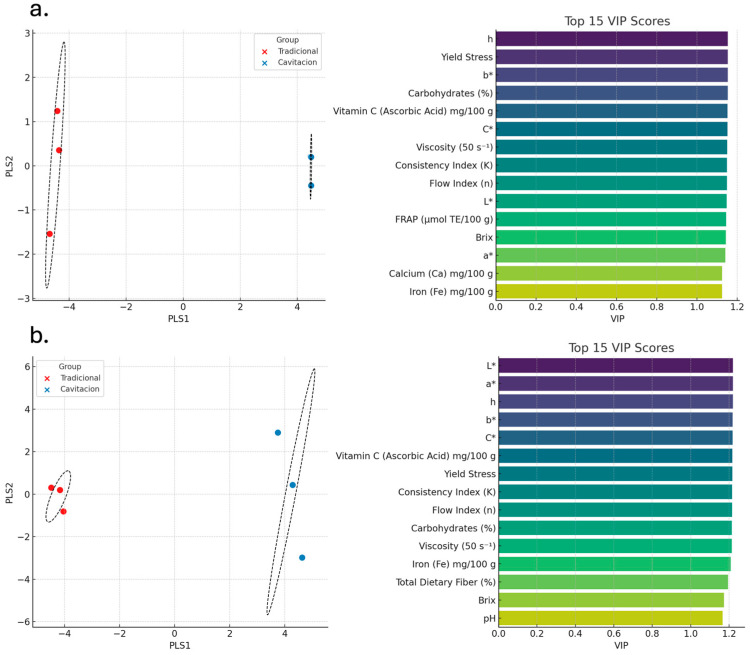
PLS-DA plots showing separation of carrot purees (cavitation/traditional) and VIP scores for relevant physicochemical and bioactive variables. (**a**) PLS-DA score plot and top 15 VIP scores for the purée samples. (**b**) PLS-DA score plot and top 15 VIP scores for the validation set.

To further interpret the data structure and sample discrimination, a Partial Least Squares Discriminant Analysis (PLS-DA) was conducted (Figure 6). The first two latent components separated purees distinctly according to treatment (cavitation vs. thermal), with the highest variable importance observed for FRAP, DPPH, and L* color coordinate (VIP > 1), highlighting them as key discriminators in the processing method.

Together, these analyses demonstrate that hydrodynamic cavitation, compared to classical thermal processing, achieves not only substantial retention of bioactive compounds and vibrant color, but also maintains superior structural and sensory qualities, thereby offering a robust, scalable strategy for the valorization of vegetable surpluses into high-value functional foods.

### 3.3. Targeted Metabolomic Profiling of Phenolic Compounds by LC–QqQ–MS

**Figure 7:**
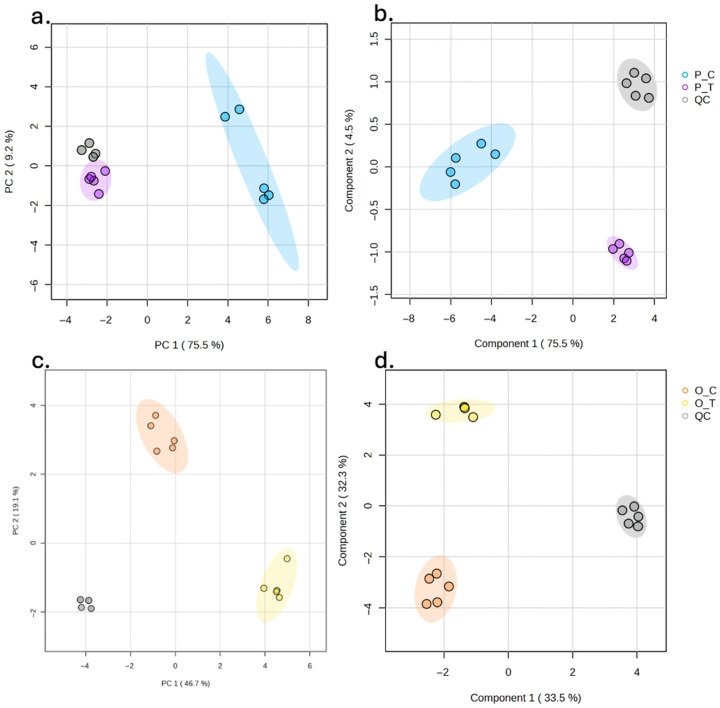
Multivariate analysis of the targeted phenolic metabolite profile of carrot-based purées processed by hydrodynamic cavitation or conventional thermal treatment. (**a**) PCA score plot for purple carrot purées (PERMANOVA: R^2^ = 0.73816, *p* < 0.001). (**b**) PLS-DA score plot for purple carrot purées (*p* < 0.01; R^2^: 0.82; Q^2^: 0.78). (**c**) PCA score plot for orange carrot purées (PERMANOVA: R^2^ = 0.98742, *p* < 0.001). (**d**) PLS-DA score plot for orange carrot purées (*p* < 0.01 R^2^: 0.98; Q^2^: 0.93). Quality control (QC) injections are shown in grey.

Multivariate analysis of the targeted phenolic metabolite profiles revealed a marked effect of both carrot type and processing technology (Figure 7). For purple carrot purées, the PCA model accounted for most of the variance in the first two principal components (PC1 = 75.5% and PC2 = 9.2%), clearly separating samples processed by hydrodynamic cavitation from those subjected to conventional thermal treatment, while QC injections clustered tightly, confirming analytical robustness.

This separation was statistically supported by PERMANOVA (R^2^ = 0.738, *p* < 0.001), indicating that approximately 74% of the multivariate variability was attributable to the processing technology. Consistently, the supervised PLS-DA model for purple carrot purées showed an even sharper discrimination between treatments, with high goodness-of-fit and predictive ability (R^2^ = 0.82, Q^2^ = 0.78, *p* < 0.01), demonstrating that hydrodynamic cavitation and thermal processing generated distinct phenolic fingerprints in this matrix. A similar pattern was observed for orange carrot purées, although with an even more pronounced separation between technologies. The PCA explained 46.7% and 16.1% of the total variance in PC1 and PC2, respectively, and differentiated clearly between hydrodynamically cavitated and thermally treated samples, with QC samples again forming a compact cluster. PERMANOVA yielded R^2^ = 0.987 and *p* < 0.001, indicating that virtually all of the metabolomic variation could be ascribed to the processing effect. The corresponding PLS-DA model confirmed this strong discrimination, with very high R^2^ (0.98) and Q^2^ (0.93) values and statistical significance (*p* < 0.01), evidencing that the phenolic profiles of orange carrot purées are particularly sensitive to the applied technology.

**Figure 8:**
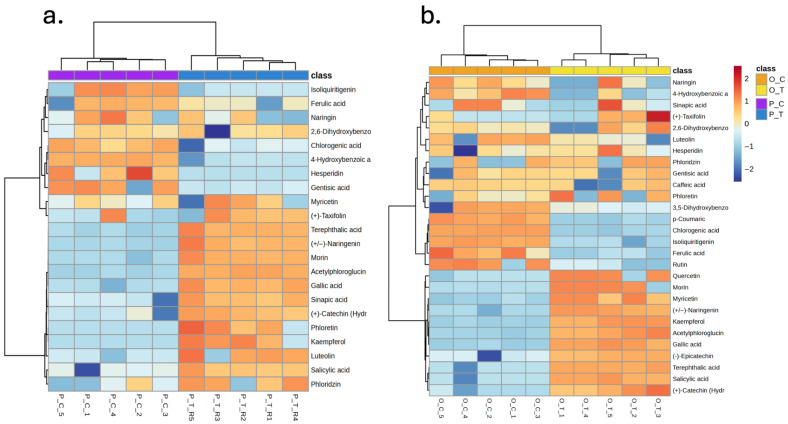
Hierarchical clustering heatmap showing differences in the abundance of targeted phenolic metabolites in carrot-based purées processed by hydrodynamic cavitation or conventional thermal treatment. (**a**) Purple carrot purées (P_C, P_T). (**b**) Orange carrot purées (O_C, O_T). The *x*-axis shows the clustering of all purée, whereas the *y*-axis shows the clustering of individual phenolic compounds. Each colored cell corresponds to a normalized (z-scaled) log-response value of metabolite levels, with samples in columns and metabolites in rows. Sample classes are indicated by the color code at the top of each heatmap.

The heatmap for purple carrot purées (Figure 8a) shows a coherent, technology-driven partition of metabolites. Samples processed by hydrodynamic cavitation (P_C) display a relative enrichment of flavanone glycosides (e.g., naringin, hesperidin) together with signature hydroxycinnamic acids such as chlorogenic and ferulic acids, and the chalcone isoliquiritigenin. In contrast, thermal treatment (P_T) is associated with higher abundances of several flavonoid aglycones—notably (+/−)-naringenin, kaempferol, luteolin, phloretin, myricetin and (+)-taxifolin—as well as simple phenolic acids from the hydroxybenzoic and hydroxycinnamic families (gallic, 4-hydroxybenzoic, salicylic, sinapic) and related phenolics (phloridzin, (+)-catechin (hydrate), acetylphloroglucinol).

Taken together, these patterns indicate that hydrodynamic cavitation preserves and/or extracts conjugated phenolics (glycosides and esterified cinnamates) under milder thermal stress, whereas conventional heating promotes deglycosylation and de-esterification (increasing aglycones and simpler benzoic/cinnamic acids) and likely oxidation/rearrangement of more complex structures. This technology-specific redistribution of chemical families aligns with the multivariate results (PCA/PLS-DA), supporting a distinct phenolic fingerprint for HC versus TT in the purple carrot matrix.

Regarding the heatmap for orange carrot purées (Figure 8b) it reveals a pronounced divergence in phenolic profiles between hydrodynamic cavitation (O_C) and thermal processing (O_T), with a stronger technology-driven clustering than that observed for purple carrot. Hydrodynamic cavitation was associated with higher relative abundances of several hydroxycinnamic acids, including *p*-coumaric, chlorogenic, and caffeic acids, compounds typically linked to antioxidant capacity, cell-wall esterification, and color stabilization in carrot matrices. Cavitated samples also exhibited elevated levels of the flavonol glycoside rutin and the chalcone isoliquiritigenin, suggesting enhanced extraction or improved preservation of glycosylated and conjugated phenolics under cavitation-driven mechanical forces and reduced thermal exposure. In contrast, thermally treated orange carrot purées (O_T) showed enrichment in multiple flavonoid aglycones, particularly kaempferol, quercetin, luteolin, phloretin, and (±)-naringenin, along with simple phenolic acids such as gallic acid, 4-hydroxybenzoic acid, and sinapic acid. The accumulation of these aglycones and low-molecular-weight benzoic/cinnamic derivatives is consistent with heat-induced deglycosylation, ester hydrolysis, and oxidation of more complex phenolic structures, processes known to occur during prolonged thermal exposure. The presence of phloridzin, acetylphloroglucinol, and (–)-epicatechin at higher levels in O_T further supports a shift toward degradation products or intermediates typically formed under thermal stress. Collectively, these results indicate that hydrodynamic cavitation preferentially preserves or enhances conjugated phenolic species, particularly glycosylated flavonoids and esterified hydroxycinnamates, while thermal treatment promotes breakdown into simpler aglycones and benzoic/cinnamic acids. This differential behavior underscores the sensitivity of orange carrot phenolic networks to processing intensity and highlights the capacity of cavitation to maintain a more structurally complex and potentially more fingerprint bioactive phenolic compared to conventional heat-based methods.

**Figure 9:**
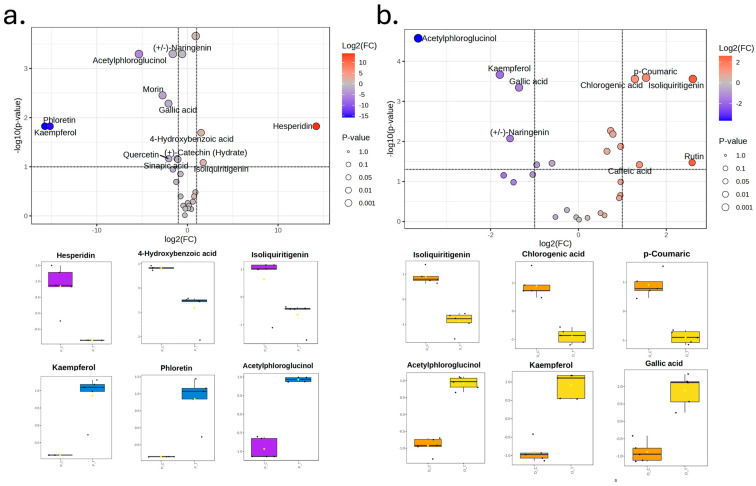
Differential accumulation of targeted phenolic metabolites in carrot-based purées processed by hydrodynamic cavitation or conventional thermal treatment. (**a**) Volcano plot and boxplots of selected discriminant metabolites for purple carrot purées, showing the relationship between log_2_ (fold change) and –log_10_ (*p*-value) for each compound, and the relative abundances of the most significantly altered phenolics between technologies. (**b**) Volcano plot and boxplots for orange carrot purées, illustrating the same comparison between processing technologies. Dotted lines in the volcano plots indicate the thresholds used to define significantly different metabolites.

The volcano plot for purple carrot purées (Figure 9a) confirms the metabolic divergences detected by PCA and PLS-DA, with hesperidin standing out as the main discriminatory metabolite, showing much higher abundance in cavitated samples (log_2_FC > 10, *p* < 0.001). Consistent with the heatmap, other conjugated phenolics such as isoliquiritigenin and chlorogenic acid were also enriched in P_C purées, indicating that hydrodynamic cavitation favors the preservation or extraction of glycosylated and esterified flavonoids. In contrast, kaempferol, phloretin, acetylphloroglucinol, and 4-hydroxybenzoic acid were significantly higher in thermally processed purées (log_2_FC < –5, *p* < 0.01), reflecting heat-induced deglycosylation, ester cleavage, and oxidative degradation. Overall, cavitation maintains a more structurally complex phenolic profile, whereas thermal treatment promotes the formation of simpler aglycones and hydroxybenzoic/hydroxycinnamic acids.

Similarly, the volcano plot for orange carrot purées (Figure 9b) highlights higher levels of *p*-coumaric, chlorogenic, and caffeic acids, rutin, and isoliquiritigenin in cavitated samples (log_2_FC > 2.0, *p* < 0.01), in line with the enrichment of conjugated hydroxycinnamates and glycosylated flavonoids in O_C purées. Thermally treated samples instead accumulated acetylphloroglucinol, kaempferol, gallic acid, and (±)-naringenin (log_2_FC < –2.0, *p* < 0.01), consistent with phenolic fragmentation and deglycosylation during heating. These trends support the conclusion that hydrodynamic cavitation preserves a more complex and bioactive phenolic matrix, whereas conventional thermal processing shifts the profile toward simpler degradation products.

## 4. Discussion

This study addresses a critical knowledge gap in food processing by examining the application of hydrodynamic cavitation (HC) to complex, multi-component fruit and vegetable matrices, evaluating its performance against conventional thermal treatment (TT). While HC has demonstrated remarkable efficacy in homogenizing low-viscosity liquid products through intense shear forces and bubble collapse phenomena [24], substrate-dependent limitations emerged when applied to fiber-rich, polydisperse carrot-based puree systems.

This mechanism theoretically offers dual benefits: cellular disruption for particle size reduction and bioactive preservation through minimal bulk thermal exposure. However, the carrot purees (total dietary fiber 2.7–3.1%) subjected to HC at operational parameters (maximum temperature 85 °C, working pressure 5 PSI, residence time 5 min) displayed markedly higher median grumoseness (HC: 7.0–8.0 vs. TT: 5.0–6.0) and fibrosidad (HC: 4.0–6.0 vs. TT: 2.0–5.0), indicating insufficient microstructural disruption. Sensory evaluations corroborated these observations, with significantly higher oral grumosiness and fibrosidad in HC-treated samples. This heterogeneous texture suggests that insoluble fiber from carrot, beetroot, and yacón, components of high mechanical resilience, resists disruption under the applied cavitation regime. It is also highlighted that the fruits used were processed with the peel, a parameter that helps to reduce waste from this process but which could influence the texture and fiber present. Castro-Muñoz et al. (2023) [24] emphasized that fiber-rich substrates show greater resistance to mechanical disruption than expected, with the mechanical energy distributed across competing processes rather than converging toward uniform homogenization. The rheological profiles confirmed significant variation among formulations (*p*-values < 0.05), yet macroscopic texture indicated HC-induced heterogeneity rather than the desired smoothness, contrasting sharply with literature on lower-viscosity systems where HC improved stability and rheological properties [24,25,26]. Despite texture limitations, HC demonstrated pronounced advantages in preserving heat-labile bioactive compounds. Purple carrot puree treated by HC achieved mean FRAP values of 2580 ± 126 μmol Eq-Trolox/100 g versus 993 ± 110 μmol Eq-Trolox/100 g in TT samples (*p*-values < 0.001) a 160% superiority. This substantial difference aligns with elevated sensory astringency in HC samples (median 5.0 vs. TT: 2.0), indicating higher retention of non-degraded phenolic compounds. DPPH inhibition similarly favored HC, with maximum values of 88.72% ± 0.80%, and synergistic mixture effects particularly pronounced for interactions involving beetroot and gulupa (*p*-values = 0.0001–0.0429). Ishida and Chapman (2012) demonstrated that hydrodynamic processes increased carotenoid extractability from tomato by 43% through mechanical membrane disruption, improving bioavailability [27]. Similarly, Arya et al. (2021) showed that HC processing in orange juice achieved 65% PME inactivation while maintaining ascorbic acid and phenolic stability, underscoring HC’s dual enzymatic control and bioactive preservation capacity [28].

Vitamin C retention proved particularly striking: HC-treated purple carrot puree retained 6.8 ± 0.6 mg/100 g versus only 0.6 ± 0.0 mg/100 g in TT samples (*p*-values < 0.001), representing a 1.033% differential that reflects ascorbic acid’s extreme heat sensitivity and HC’s effectiveness at minimizing oxidative degradation. Orange carrot puree exhibited similar patterns (HC: 2.4 ± 0.1 mg/100 g vs. TT: 0.8 ± 0.0 mg/100 g). Mineral content (Fe, Ca, Zn) remained stable across treatments (*p*-values > 0.05), indicating that the thermo-hydrodynamic mechanism does not induce leaching or chemical transformation of inorganic micronutrients a favorable outcome consistent with other non-thermal technologies [29]. HC also preserved characteristic vegetable flavors more effectively than TT. Sensory profiling revealed that HC retained significantly more intense carrot flavor (median 6.0 vs. TT: 2.0, *p* = 0.014 for purple carrot; 4.0 vs. TT: 2.0, *p* = 0.025 for orange carrot) and earthy notes, demonstrating preservation of authentic sensory identity through retention of volatile compounds such as terpenes and sulfur-containing compounds (Barba et al., 2020 [25]). Swiacka et al. (2025) [30] similarly documented that ultrasound-assisted treatments retained vegetable-characteristic aromas better than conventional methods, supporting the principle that non-thermal mechanical processing minimizes degradation of heat-sensitive volatiles. Sabahi et al. (2024) further reported that ultrasound-assisted extraction from carrot pomace yielded higher antioxidant activity through cell wall permeabilization, suggesting HC operates analogously to enhance compound accessibility and stability in multi-component systems [30,31].

Paradoxically, despite superior bioactive retention and authentic flavor preservation, HC-treated purees exhibited significantly lower overall acceptance than TT samples (purple carrot HC: 1.0 [0.0–2.0] vs. TT: 3.0 [3.0–4.0], *p* = 0.011; orange carrot HC: 2.0 [1.0–3.0] vs. TT: 4.0 [3.0–4.0], *p*-values = 0.033). This inverse relationship highlights a central challenge in functional food development: balancing nutritional superiority with sensory hedonic quality. Thermal treatment generated significantly more intense fruity notes—particularly banana flavor (TT: 7.0 vs. HC: 4.0, *p*-values = 0.014 for purple carrot; TT: 6.0 vs. HC: 2.0, *p*-values = 0.013 for orange carrot)—and enhanced perceived sweetness (TT: 5.0 vs. HC: 3.0), likely through ester formation, polysaccharide hydrolysis to reducing sugars, Maillard reactions, and caramelization. These thermally induced modifications, while reducing bioactivity through phenolic degradation and vitamin oxidation, enhanced hedonic perception by masking vegetable bitterness and astringency with desirable caramel and fruity notes. The textural roughness of HC samples, elevated grumoseness and fibrosidad, combined with preserved vegetable, earthy, and phenolic astringency notes, created a sensory profile perceived as less refined than the smoother, sweeter, fruit-forward profile of TT products.

Partial Least Squares Discriminant Analysis identified FRAP, DPPH inhibition, and luminosity (L*) as highest-ranked discriminators (VIP > 1.0) between treatments, confirming that HC versus TT represents a fundamental trade-off between antioxidant functionality and sensory acceptance rather than unequivocal technological superiority. The optimization achieving desirability scores of 1.00 for both carrot types (purple: 46% carrot, 40% banana, 7% yacón, 3% beetroot, 4% gulupa; orange: 51% carrot, 35% banana, 5% yacón, 4% beetroot, 5% gulupa) demonstrated that multi-component blending partially mitigated texture limitations by incorporating smoother-textured components with high soluble solids. However, absolute fiber content remained limiting under current HC regimens, with residual grumoseness persisting despite careful formulation, indicating that reactor-level parameter tuning is essential. To transition HC from a bioactive-preserving technology to a comprehensive functional product platform, several strategies warrant investigation. Parametric optimization increasing working pressure (10–20 PSI) and/or residence time (10–15 min) could enhance shear-induced disruption without proportionally increasing bulk temperature. Multi-pass processing analogous to multi-stage in high-pressure homogenization protocols might achieve cumulative particle size reduction without excessive thermal load. Hurdle strategies integrating HC with complementary non-thermal technologies (pulsed light, cold atmospheric plasma) could achieve synergistic cell disruption while mitigating adverse texture changes [26]. Post-HC biopolymer supplementation (pectin, xanthan gum) could normalize texture perception without additional processing, decoupling sensory quality from inherent matrix properties. Targeted flavor enhancement using natural vegetable-compatible compounds could augment HC product sensory profiles toward greater acceptability without compromising demonstrated nutritional advantages.

The preservation of bioactives through HC, despite texture trade-offs, underscores its strategic value for valorizing surplus fruits and vegetables into functional ingredients rather than finished products. While HC-treated purees may not compete in mainstream consumer markets given lower hedonic ratings, they represent superior functional ingredients for supplementation into complementary foods, fortified beverages, solid dosage forms, and plant-based meat and dairy alternatives where antioxidant fortification is valued. This study, conducted on surplus materials from REAGRO/Colombia Foodbank and Agrosavia programs, directly addresses food waste mitigation and nutritional security objectives aligned with ONU Sustainable Development Goals. By demonstrating HC’s superior bioactive retention and authentic sensory preservation alongside candid assessment of textural challenges and acceptability trade-offs, this research provides a foundation for future investigations into optimized processing parameters and integrative strategies that could reconcile functional superiority with sensory quality in valorized vegetable-based products.

Regarding metabolic differences, the marked divergence in phenolic profiles between hydrodynamically cavitated and thermally processed carrot purées reflects distinct biochemical transformations imposed by each technology. Cavitation-treated samples preferentially retain glycosylated flavonoids (hesperidin, rutin, naringin) and esterified hydroxycinnamic acids (chlorogenic, caffeic, *p*-coumaric), whereas thermally processed purées accumulate flavonoid aglycones (kaempferol, quercetin, luteolin, naringenin, phloretin) and simple phenolic acids (gallic, 4-hydroxybenzoic, sinapic) [2,32]. Hydrodynamic cavitation promotes cell disruption and phenolic release mainly through intense mechanical forces, microjets, shear stress, and rapid pressure fluctuations—that break cell walls and weaken matrix, phenolic interactions while limiting overall thermal exposure. This mechanical mode of action increases the extractability of structurally complex conjugated phenolics without providing the sustained time–temperature conditions needed to drive extensive glycosidic or ester bond hydrolysis, so glycosylated flavonoids and hydroxycinnamate esters are released largely intact into the soluble fraction, preserving their native structural complexity and associated bioactivity [33].

In contrast, conventional thermal treatment imposes prolonged exposure to elevated temperatures, creating conditions that favor multiple degradative pathways. Heat-induced deglycosylation of flavonoid glycosides proceeds via both enzymatic and nonenzymatic mechanisms: endogenous glycosidases (e.g., rutin-degrading enzymes) can cleave glycosidic bonds before thermal inactivation occurs, particularly under moist-heat conditions where enzyme activity persists at temperatures up to approximately 50–60 °C [34,35] at higher temperatures, direct thermal or acid/base-catalyzed hydrolysis of O-glycosidic bonds becomes the dominant mechanism, yielding free aglycones and reducing sugars [33]. Similarly, esterified hydroxycinnamic acids, most notably chlorogenic acid (5-O-caffeoylquinic acid)—undergo thermal hydrolysis to release caffeic acid and quinic acid, a reaction that accelerates with increasing temperature and in aqueous or acidic environments [36]. The elevated levels of caffeic acid, *p*-coumaric acid, and other simple hydroxycinnamic derivatives observed in thermally treated samples are consistent with this ester cleavage mechanism. Furthermore, prolonged heating promotes oxidative degradation of released phenolic aglycones and acids, generating phenoxyl radicals and quinones that can undergo irreversible polymerization, condensation, or Maillard-type reactions with amino groups, thereby reducing the concentration of extractable monomeric phenolics and forming higher-molecular-weight, less bioavailable compounds [37].

The accumulation of simple hydroxybenzoic acids (gallic, 4-hydroxybenzoic) in thermally processed samples likely arises from secondary fragmentation of more complex phenolic structures under oxidative and thermal stress, including ring fission, decarboxylation, and hydrolytic cleavage of C–C bonds in flavonoid skeletons [38]. These transformations are temperature- and time-dependent, with kinetic studies demonstrating accelerated degradation rates above 70–80 °C and significant losses during extended pasteurization or sterilization regimes [39]. The matrix-specific sensitivity observed for orange carrot purées—reflected in the nearly complete separation of phenolic profiles between processing technologies (PERMANOVA R^2^ = 0.987)—may be attributed to differences in endogenous enzyme activity, cell wall composition, or the initial distribution of bound versus free phenolics compared to purple carrot matrices [40].

These results underscore that hydrodynamic cavitation operates within a “preservation window” where mechanical disruption maximizes phenolic extractability without crossing the thermal threshold required for extensive hydrolysis and oxidation, thereby maintaining a structurally complex and potentially more bioactive phenolic fingerprint. In contrast, conventional thermal processing inevitably shifts the phenolic equilibrium toward simpler, degraded forms—a trade-off inherent to heat-based stabilization strategies. The distinct biochemical trajectories observed here highlight the potential of hydrodynamic cavitation as a processing technology to preserve the native phytochemical complexity of plant-based foods, with direct implications for the functional quality and health-promoting attributes of carrot-based purées and related products.

## 5. Conclusions

Hydrodynamic cavitation (HC) proved to be an effective non-thermal technology for valorizing fruit and vegetable surpluses, achieving the primary objective of maximizing nutritional and functional quality relative to conventional thermal treatment (TT). In purple carrot purées, HC retained vitamin C at 6.8 ± 0.6 mg/100 g versus 0.6 ± 0.0 mg/100 g in TT (≈1.033% higher), and FRAP antioxidant capacity was 2.5-fold greater (2580 ± 126 vs. 993 ± 110 μmol Eq-Trolox/100 g), with DPPH inhibition reaching 88.72% ± 0.80%. Targeted metabolomics supported these results by showing that HC preserved a more structurally complex phenolic profile, with higher levels of conjugated flavonoids and hydroxycinnamic derivatives, whereas TT favored the formation of simpler phenolic acids and aglycones, indicating more extensive degradation of native metabolites. Despite these biochemical advantages, HC-treated samples exhibited higher grumosity (median 7.0–8.0 vs. 5.0–6.0) and fibrosity (4.0–6.0 vs. 2.0–5.0), leading to lower overall acceptance (1.0–2.0 vs. 3.0–4.0, *p* < 0.05). This establishes a clear trade-off: HC optimizes nutritional and metabolomic quality but compromises texture. Optimization strategies—such as fine-tuning pressure and residence time, multi-pass cavitation, and post-processing structuring or biopolymer incorporation—are therefore required to mitigate sensory limitations while retaining HC’s functional benefits. In this context, HC emerges as a viable platform for generating phenolic-rich, value-added ingredients from agri-food surpluses, contributing to food waste reduction, circular bioeconomy goals, and nutritional security in resource-limited settings.

## Figures and Tables

**Table 1:** ANOVA summary: model *p*-values, significant mixture terms (*p*), and R^2^ for color, viscosity, and antioxidant capacity of purple and orange carrot purees.

Source	Response Variables for Formulation of Purple Carrot							Response Variables for Formulation of Orange Carrot						
	L*	a*	b*	Viscosity	DPPH	TPC	FRAP	L*	a*	b*	Viscosity	DPPH	TPC	FRAP
	*p*-Values	*p*-Values	*p*-Values	*p*-Values	*p*-Values	*p*-Values	*p*-Values	*p*-Values	*p*-Values	*p*-Values	*p*-Values	*p*-Values	*p*-Values	*p*-Values
Model	<0.0001	<0.0001	<0.0001	<0.0001	<0.0001	<0.0001	0.0049	<0.0001	0.0047	<0.0001	0.0229	<0.0001	<0.0001	<0.0001
^(1)^ Linear Mixture	<0.0001	<0.0001	<0.0001	<0.0001	<0.0001	<0.0001	0.0465	<0.0001	0.0019	<0.0001	0.0376	<0.0001	<0.0001	<0.0001
AB	0.2188	0.7806	0.4977	------	------	------	0.0138	------	0.0043	0.7988	0.3680	------	------	------
AC	0.4698	0.3886	0.9994	0.3973	------	------	0.3430	0.5863	0.2654	------	0.3930	------	0.8283	0.7846
AD	<0.0001	0.0009	<0.0001	<0.0001	0.1342	0.1411	------	0.4388	------	<0.0001	0.2713	0.0292	0.4339	0.0125
AE	0.4227	0.0094	0.0402	0.5256	0.8645	0.1372	0.2675	0.0039	------	0.0009	0.3955	------	0.9500	0.7785
BD	0.0098	0.9860	0.0006	<0.0001	------	------	------	------	------	0.0078	0.7577	------	------	------
BE	------	0.8768	------	0.4336	------	------	0.1906	------	------	------	0.1498	------	------	------
BC	------	0.4719	------	------	------	------	0.2526	------	0.2847	------	0.5895	------	------	------
CD	0.0290	0.2528	0.3196	0.0027	------	0.0429		0.3951	0.1593	------	0.1249	------	------	0.0029
CE	0.7196	0.6314	------	0.7424	------	------	0.7484	------	0.2492	------	0.6387	------	0.4245	0.9870
DE	<0.0001	0.0529	0.0261	<0.0001	0.3866	0.2429	0.0016	------	0.6276	------	0.6411	0.0222	0.1522	0.5858
ACD	0.0024	------	0.0021	------	------	------	------	0.0142	------	------	0.0302	------	------	------
ABC	------	------	------	------	------	------	------	------	0.0408	------	0.0130	------	------	------
CDE	------	------	------	------	------	------	------	------	0.3703	------	0.0178	------	------	0.0353
ABD	0.0003	0.0104	0.0018	------	------	------	------	------	------	0.0578	0.0221	------	------	------
ADE	------	0.0769	0.1103	<0.0001	0.0634	0.0002		------	------	------	0.1951	------	0.0181	------
ACE	0.0004	0.0003	------	<0.0001	------	------	0.0243	------	------	------	------	------	0.0160	0.0865
BCD	------	0.0133	------	------	------	------	------	------	------	------	0.1523	------	------	------
BDE	------	0.0222	------	0.0367	------	------	------	------	------	------	------	------	------	------
CDE	------	0.0004	------	------	------	------	------	------	------	------	------	------	------	------
ABE	------	------	------	------	------	------	0.0020	------	------	------	------	------	------	------
BCE	------	------	------	------	------	------	0.1736	------	------	------	------	------	------	------
R^2^	0.9525	0.9696	0.9348	0.9478	0.9224	0.9458	0.7772	0.9317	0.7391	0.9182	0.8752	0.7706	0.9764	0.9741
Adj. R^2^	0.9050	0.9088	0.8778	0.8956	0.8942	0.9226	0.5822	0.8975	0.5549	0.8832	0.6255	0.7108	0.9619	0.9558

^(1)^ Linear Mixture: linear effect of the component proportions in a mixture design; (----) indicates non-significant terms removed during model selection. A: Carrot, B: Banana, C: Yacon, D: Beetroot, E: Gulupa, Viscosity: expressed in cP, DPPH expressed in Inhibition (%), TPC expressed in mg EAG/g, FRAP expressed in µmol Eq-Trolox/100 g.

## Data Availability

The original contributions presented in this study are included in the article/Supplementary Materials. Further inquiries can be directed to the corresponding authors.

## Supplementary Materials

The following supporting information can be downloaded at: https://www.mdpi.com/article/10.3390/foods15020268/s1, Table S1: Experimental matrix and measured response variables for purple and orange carrot puree formulations; Table S2: Summary of mean values (± SD) and Tukey groupings (α = 0.05) for physicochemical and functional parameters obtained from carrot-based products processed under traditional and hydrodynamic cavitation technologies; Table S3: MRM conditions for the semi-quantification of phenolic compounds and flavonoids in positive and negative polarity by LC-QqQ.

## Abbreviations

The following abbreviations are used in this manuscript:

HC	Hydrodynamic cavitation
TT	Thermal treatment
TPC	Total polyphenolic content (contenido fenólico total)
FRAP	Ferric Reducing Antioxidant Power
DPPH	2,2-diphenyl-1-picrylhydrazyl (ensayo de capacidad antioxidante)
AOAC	Association of Official Analytical Chemists
ANOVA	Analysis of Variance
LOOCV	Leave-One-Out Cross-Validation
PLS-DA	Partial Least Squares Discriminant Analysis
SDG/ODS	Sustainable Development Goals/Objetivos de Desarrollo Sostenible
CIELab	Sistema internacional de colorimetría
ORAC	Oxygen Radical Absorbance Capacity
VIP	Variable Importance in Projection
