# Supplementary material for: Non-Thermal Hydrodynamic Cavitation for Surplus Fruits and Vegetables: Improved Vitamin C and Bioactive Preservation
Source: Foods. 2026 Jan 12;15(2):268. doi: 10.3390/foods15020268 (PMC12839826; doi:10.3390/foods15020268)
Supplement: Supplementary file 1 [file foods-15-00268-s001.zip › foods-4013024-supplementary.pdf]

Table S1. Experimental matrix and measured response variables for purple and orange carrot puree formulations.

A	B	C	D	E	Response variables for formulation of purple carrot							Response variables for formulation of orange carrot						
					L*	a*	b*	Viscosity	DPPH	TPC	FRAP	L*	a*	b*	Viscosity	DPPH	TPC	FRAP
1.00	0.00	0.00	0.00	0.00	14.58 (± 1.84)	8.01 (± 6.66)	6.21 (± 0.91)	26850 (± 127.28)	78.10 (± 0.60)	0.40 (± 0.02)	242.67 (± 5.89)	43.94 (± 0.23)	26.61 (± 7.25)	38.93 (± 0.07)	34150 (± 2701.15)	49.53 (± 0.50)	0.17 (± 0.01)	61.07 (± 6.63)
0.00	1.00	0.00	0.00	0.00	32.67 (± 0.18)	7.61 (± 0.08)	21.99 (± 0.09)	39330 (± 2163.75)	88.25 (± 0.20)	0.31 (± 0.02)	183.99 (± 3.68)	32.67 (± 0.18)	7.61 (± 0.08)	21.99 (± 0.09)	39330 (± 2163.75)	88.25 (± 0.20)	0.31 (± 0.02)	183.99 (± 3.68)
0.00	0.00	1.00	0.00	0.00	20.33 (± 0.21)	-1.23 (± 0.07)	6.51 (± 0.03)	26190 (± 2078.89)	82.46 (± 4.42)	0.26 (± 0.0)	167.32 (± 2.21)	20.33 (± 0.21)	-1.23 (± 0.07)	6.51 (± 0.03)	26190 (± 2078.89)	82.46 (± 4.42)	0.26 (± 0.0)	167.32 (± 2.21)
0.00	0.00	0.00	1.00	0.00	17.48 (± 1.21)	10.85 (± 0.36)	5.43 (± 0.14)	35790 (± 1315.22)	80.28 (± 0.40)	1.44 (± 0.04)	741.28 (± 2.95)	17.48 (± 1.21)	10.85 (± 0.36)	5.43 (± 0.14)	35790 (± 1315.22)	80.28 (± 0.40)	1.44 (± 0.04)	741.28 (± 2.95)
0.00	0.00	0.00	0.00	1.00	34.505 (± 0.01)	25.48 (± 0.04)	20.61 (± 0.20)	30600 (± 7636.75)	83.79 (± 1.61)	1.99 (± 0.05)	887.11 (± 2.95)	34.51 (± 0.01)	25.48 (± 0.04)	20.61 (± 0.20)	30600 (± 7636.75)	83.79 (± 1.61)	1.99 (± 0.05)	887.11 (± 2.95)
0.50	0.50	0.00	0.00	0.00	28.17 (± 0.01)	6.35 (± 0.06)	12.21 (± 0.05)	50280 (± 4582.05)	72.84 (± 1.71)	0.56 (± 0.02)	354.82 (± 10.31)	40.49 (± 2.07)	40.49 (± 2.07)	30.97 (± 0.16)	40710 (± 3181.98)	85.12 (± 1.61)	0.24 (± 0.0)	141.80 (± 6.63)
0.50	0.00	0.50	0.00	0.00	20.38 (± 0.25)	1.43 (± 0.02)	7.59 (± 0.07)	15350 (± 777.82)	79.15 (± 1.21)	0.28 (± 0.02)	174.61 (± 4.42)	32.80 (± 0.23)	7.28 (± 0.40)	21.11 (± 0.74)	37160 (± 1725.34)	69.67 (± 4.62)	0.19 (± 0.02)	127.74 (± 0.74)
0.50	0.00	0.00	0.50	0.00	34.80 (± 0.66)	20.06 (± 0.82)	27.22 (± 0.89)	43740 (± 1442.50)	43.22 (± 2.41)	0.58 (± 0.03)	406.91 (± 0.74)	15.84 (± 2.06)	6.17 (± 0.48)	3.67 (± 0.31)	48120 (± 2885.00)	63.03 (± 0.40)	0.34 (± 0.02)	279.82 (± 13.26)
0.50	0.00	0.00	0.00	0.50	28.18 (± 0.60)	8.54 (± 0.01)	9.75 (± 0.07)	38040 (± 8315.58)	82.56 (± 0.20)	1.32 (± 0)	706.91 (± 10.31)	27.55 (± 0.17)	17.08 (± 1.64)	15.02 (± 0.31)	38040 (± 8315.58)	87.39 (± 0.20)	1.03 (± 0.02)	601.70 (± 2.95)
0.00	0.50	0.50	0.00	0.00	29.315 (± 0.13)	1.13 (± 0.04)	11.66 (± 0.01)	35970 (± 127.28)	88.72 (± 0.80)	0.31 (± 0.03)	237.11 (± 8.84)	29.32 (± 0.13)	1.13 (± 0.04)	11.66 (± 0.01)	35970 (± 127.28)	88.72 (± 0.80)	0.31 (± 0.03)	237.11 (± 8.84)
0.00	0.50	0.00	0.5	0.00	19.32 (± 0.03)	9.29 (± 0.01)	4.15 (± 0.01)	36300 (± 763.68)	45.02 (± 1.61)	0.48 (± 0.0)	456.91 (± 72.18)	19.32 (± 0.03)	9.29 (± 0.01)	4.15 (± 0.01)	36300 (± 763.68)	45.02 (± 1.61)	0.48 (± 0.0)	456.91 (± 72.18)
0.00	0.50	0.00	0.00	0.50	36.04 (± 0.02)	15.78 (± 0.06)	19.27 (± 0.02)	43250 (± 862.67)	88.20 (± 0.50)	1.20 (± 0.02)	639.20 (± 8.84)	36.04 (± 0.02)	15.78 (± 0.06)	19.27 (± 0.02)	43250 (± 862.67)	88.20 (± 0.50)	1.20 (± 0.02)	639.20 (± 8.84)
0.00	0.00	0.50	0.50	0.00	13.32 (± 1.61)	2.92 (± 0.12)	2.81 (± 0.01)	16320 (± 424.26)	60.90 (± 0.10)	0.40 (± 0.01)	303.09 (± 11.79)	13.32 (± 1.61)	2.92 (± 0.12)	2.81 (± 0.01)	16320 (± 424.26)	60.90 (± 0.10)	0.40 (± 0.01)	303.09 (± 11.79)
0.00	0.00	0.50	0.00	0.50	12.71 (± 0.75)	7.43 (± 0.04)	4.23 (± 0.08)	8520 (± 2425.38)	84.55 (± 0.40)	1.18 (± 0.04)	643.36 (± 1.47)	28.75 (± 0.75)	11.49 (± 0.04)	13.76 (± 0.08)	26015 (± 2425.38)	84.55 (± 0.40)	1.18 (± 0.04)	643.36 (± 1.47)
0.00	0.00	0.00	0.50	0.50	14.46 (± 2.81)	16.32 (± 0.95)	6.97 (± 1.89)	46200 (± 1272.79)	28.48 (± 0.30)	1.45 (± 0.02)	873.57 (± 48.61)	13.89 (± 2.81)	13.39 (± 0.95)	6.90 (± 1.89)	34800 (± 1272.79)	28.48 (± 0.30)	1.45 (± 0.02)	873.57 (± 48.61)

0.33	0.33	0.33	0.00	0.00	23.26 (± 6.75)	8.93 (± 0.13)	10.85 (± 0.31)	46230 (± 339.41)	86.26 (± 1.01)	0.33 (± 0.0)	211.07 (± 10.31)	22.33 (± 6.75)	5.59 (± 0.13)	19.01 (± 0.31)	19680 (± 339.41)	90.62 (± 0.20)	0.23 (± 0.02)	172.53 (± 5.89)
0.33	0.33	0.00	0.33	0.00	17.65 (±0.32)	9.66 (± 0.06)	4.39 (± 0.01)	37200 (± 5388.15)	40.95 (± 0.80)	0.60 (± 0.0)	327.74 (± 28.73)	28.34 (± 0.32)	11.36 (± 0.06)	20.87 (± 0.01)	30150 (± 5388.15)	70.90 (± 2.61)	0.29 (± 0.03)	271.84 (± 30.94)
0.33	0.33	0.00	0.00	0.33	13.32 (± 4.55)	2.92 (± 2.57)	2.81 (± 3.05)	16320 (± 1852.62)	86.35 (± 0.80)	0.82 (± 0.02)	523.92 (± 8.84)	30.67 (± 4.55)	15.67 (± 2.57)	23.19 (± 3.05)	46210 (± 1852.62)	88.06 (± 0.40)	0.92 (± 0.06)	419.75 (± 17.68)
0.33	0.00	0.33	0.33	0.00	12.71 (± 0.04)	7.43 (± 0.15)	4.23 (± 0.01)	8520 (± 424.26)	61.23 (± 3.22)	0.48 (± 0.02)	287.11 (± 17.68)	13.86 (± 0.83)	14.09 (± 0.28)	8.34 (± 0.02)	19950 (± 296.98)	72.99 (± 1.21)	0.30 (± 0.04)	176.70 (± 1.47)
0.33	0.00	0.33	0.00	0.33	23.27 (± 1.17)	9.31 (± 0.04)	11.25 (± 0.04)	47280 (± 84.85)	47.25 (± 0.60)	0.91 (± 0.0)	498.57 (± 69.24)	32.44 (± 0.71)	12.93 (± 0.41)	20.37 (± 0.56)	28740 (± 339.41)	87.01 (± 0.40)	0.99 (± 0.01)	474.61 (± 26.52)
0.33	0.00	0.00	0.33	0.33	23.25 (± 0.02)	8.74 (± 0.54)	10.65 (± 0.57)	45705 (± 1484.92)	88.82 (± 0.80)	0.71 (± 0.03)	343.36 (± 13.26)	21.98 (± 0.12)	19.38 (± 0.48)	9.46 (± 0.18)	53400 (± 848.53)	37.44 (± 0.60)	1.25 (± 0.03)	541.98 (± 36.83)
0.00	0.33	0.33	0.33	0.00	17.24 (± 3.96)	9.67 (± 0.65)	4.39 (± 4.43)	35640 (± 6013.94)	49.76 (± 0.0)	0.48 (± 0.03)	286.07 (± 6.63)	17.45 (± 0.29)	9.67 (± 0.01)	4.39 (± 0.0)	36420 (± 1103.09)	49.76 (± 0.0)	0.48 (± 0.03)	286.07 (± 6.63)
0.00	0.33	0.33	0.00	0.33	25.33 (± 5.72)	7.05 (± 1.85)	13.07 (± 6.14)	46500 (± 7679.18)	86.54 (± 1.01)	0.77 (± 0.01)	453.78 (± 27.99)	28.62 (± 4.65)	7.71 (± 0.93)	14.19 (± 1.58)	45780 (± 1018.23)	86.54 (± 1.01)	0.77 (± 0.01)	453.78 (± 27.99)
0.00	0.33	0.00	0.33	0.33	20.94 (± 3.85)	15.71 (± 8.11)	8.17 (± 3.37)	45180 (± 339.41)	58.29 (± 0.60)	1.02 (± 0.04)	651 (± 19.15)	20.42 (± 0.74)	17.12 (± 1.99)	8.24 (± 0.09)	46080 (± 1272.79)	58.29 (± 0.60)	1.02 (± 0.04)	651.00 (± 19.15)
0.00	0.00	0.33	0.33	0.33	22.61 (± 1.72)	16.92 (± 1.70)	8.08 (± 0.13)	46440 (± 1781.91)	49.24 (± 0.30)	1.15 (± 0.01)	658.64 (± 38.30)	18.15 (± 0.52)	17.22 (± 1.28)	7.94 (± 0.08)	47194 (± 715.59)	49.24 (± 0.30)	1.15 (± 0.01)	658.64 (± 38.30)
0.25	0.25	0.25	0.25	0.00	18.02 (± 0.17)	12.03 (± 0.18)	5.35 (± 0.01)	40020 (± 2630.44)	63.46 (± 0.20)	0.48 (± 0.03)	305.86 (± 4.42)	22.97 (± 0.33)	11.36 (± 0.90)	6.52 (± 0.22)	1907.50 (± 0.71)	69.10 (± 1.01)	0.40 (± 0.01)	269.75 (± 5.89)
0.25	0.25	0.25	0.00	0.25	19.82 (± 0.04)	13.98 (± 0.11)	6.30 (± 0.04)	37170 (± 4200.21)	79.62 (± 3.42)	0.77 (± 0.02)	385.03 (± 8.84)	34.03 (± 0.35)	10.80 (± 0.29)	20.44 (± 0.35)	46140 (± 1187.94)	67.20 (± 2.21)	1.06 (± 0.05)	497.53 (± 20.62)
0.25	0.25	0.00	0.25	0.25	20.75 (± 0.14)	16.15 (± 0.04)	6.79 (± 0.03)	43230 (± 636.40)	71.56 (± 0.20)	0.87 (± 0.01)	495.45 (± 20.62)	22.76 (± 0.27)	20.79 (± 0.02)	11.05 (± 0.04)	39270 (± 9036.82)	76.49 (±1.01)	0.86 (± 0.01)	432.95 (± 11.79)
0.25	0.00	0.25	0.25	0.25	19.12 (± 0.15)	18.14 (± 0.02)	9.56 (± 0.08)	39270 (± 9036.82)	69.19 (± 0.40)	0.95 (± 0.04)	506.56 (± 2.95)	16.93 (± 1.21)	23.23 (± 0.18)	11.07 (± 0.23)	40140 (± 933.38)	64.88 (± 0.50)	1.03 (± 0.02)	628.78 (± 38.30)
0.00	0.25	0.25	0.25	0.25	20.84 (± 0.11)	21.01 (± 0.51)	9.41 (± 0.27)	46710 (± 212.13)	68.06 (± 0.40)	0.99 (± 0.04)	520.45 (± 20.62)	20.84 (± 0.11)	21.01 (± 0.51)	9.41 (± 0.27)	46710 (± 212.13)	68.06 (± 0.40)	0.99 (± 0.04)	520.45 (± 20.62)
0.20	0.20	0.20	0.20	0.20	18.05 (± 1.23)	16.15 (± 0.42)	6.85 (± 0.20)	43170 (± 212.13)	61.23 (± 1.61)	0.52 (± 0.02)	319.75 (± 27.99)	20.74 (± 0.23)	17 (± 0.16)	8.86 (± 0.01)	28140 (± 424.26)	75.83 (± 1.61)	0.81 (± 0.01)	410.03 (± 22.10)

Values are expressed as mean ± standard deviation (n = 3). A: Carrot. B: Banana. C: Yacon. D: Beetroot . E: Gulupa. Viscosity: expressed in cP. DPPH expressed in Inhibition (%). TPC expressed in mg EAG/g. FRAP expressed in µmol Eq-Trolox/100 g.

Table S2. Summary of mean values (± SD) and Tukey groupings (α = 0.05) for physicochemical and functional parameters obtained from carrot-based products processed under traditional and hydrodynamic cavitation technologies.

Production Technology	Variable	Orange carrot	Purple carrot
		Mean ± SD (Tukey group)	Mean ± SD (Tukey group)
Cavitation	pH	4.93 ± 0.02 b	4.997 ± 0.006 b
Traditional		4.867 ± 0.006 a	4.83 ± 0.044 a
Cavitation	Brix	17.133 ± 0.115 b	17.667 ± 0.058 b
Traditional		15.933 ± 0.058 a	17.333 ± 0.058 a
Cavitation	Moisture (%)	82.02 ± 0.332 a	80.943 ± 0.196 ab
Traditional		83.047 ± 0.446 b	81.313 ± 0.386 ab
Cavitation	Ashes (%)	0.63 ± 0.026 ab	14.647 ± 24.555 ab
Traditional		0.57 ± 0.078 ab	0.54 ± 0.0 ab
Cavitation	Protein (%)	5.42 ± 0.02 ab	5.467 ± 0.051 ab
Traditional		5.223 ± 0.25 ab	5.48 ± 0.026 ab
Cavitation	Fat (%)	0.073 ± 0.006 ab	0.5 ± 0.347 ab
Traditional		0.07 ± 0.017 ab	0.118 ± 0.001 ab
Cavitation	Carbohydrates (%)	11.117 ± 0.035 b	11.393 ± 0.042 b
Traditional		9.303 ± 0.025 a	10.687 ± 0.032 a
Cavitation	Total Dietary Fiber (%)	2.913 ± 0.057 b	3.137 ± 0.015 b
Traditional		2.71 ± 0.01 a	3.05 ± 0.0 a
Cavitation	Vitamin C (Ascorbic Acid) mg/100 g	2.363 ± 0.067 b	6.76 ± 0.305 b
Traditional		0.757 ± 0.015 a	0.63 ± 0.0 a
Cavitation	Iron (Fe) mg/100 g	1.673 ± 0.075 b	0.653 ± 0.084 a
Traditional		0.967 ± 0.115 a	1.397 ± 0.015 b
Cavitation		6.123 ± 0.179 ab	6.237 ± 0.106 b

Traditional	Total Titratable Acidity (meq Acid/100 g)	6.117 ± 0.031 ab	6.02 ± 0.0 a
Cavitation	Calcium (Ca) mg/100 g	31.573 ± 0.767 b	28.753 ± 0.938 ab
Traditional		26.2 ± 0.7 a	29.223 ± 0.061 ab
Cavitation	Zinc (Zn) mg/100 g	0.85 ± 0.087 b	0.497 ± 0.084 ab
Traditional		0.643 ± 0.049 a	0.453 ± 0.055 ab
Cavitation	Firmness	118.043 ± 2.208 b	129.883 ± 4.618 ab
Traditional		108.78 ± 2.071 a	131.84 ± 1.058 ab
Cavitation	Consistency	347.757 ± 40.775 ab	388.563 ± 5.072 ab
Traditional		334.633 ± 6.28 ab	398.737 ± 13.855 ab
Cavitation	Cohesiveness	-83.753 ± 5.146 ab	-85.767 ± 8.985 ab
Traditional		-79.707 ± 0.769 ab	-99.283 ± 3.302 ab
Cavitation	Cohesion work	-128.287 ± 14.582 ab	-130.457 ± 22.636 ab
Traditional		-127.41 ± 2.865 ab	-129.933 ± 39.579 ab
Cavitation	L*	32.593 ± 0.015 a	17.09 ± 0.046 b
Traditional		33.363 ± 0.067 b	13.49 ± 0.01 a
Cavitation	a*	31.823 ± 0.038 a	18.043 ± 0.057 a
Traditional		32.963 ± 0.14 b	22.86 ± 0.044 b
Cavitation	b*	37.977 ± 0.023 b	11.237 ± 0.068 b
Traditional		31.157 ± 0.114 a	5.223 ± 0.108 a
Cavitation	C*	49.547 ± 0.029 b	21.257 ± 0.084 a
Traditional		45.357 ± 0.182 a	23.45 ± 0.017 b
Cavitation	h	50.033 ± 0.042 b	31.903 ± 0.091 b
Traditional		43.39 ± 0.026 a	12.873 ± 0.284 a
Cavitation	Viscosity (50 s^{-1})	3.593 ± 0.057 b	4.34 ± 0.1 b
Traditional		2.093 ± 0.064 a	2.81 ± 0.066 a
Cavitation	Flow Index (n)	0.3 ± 0.0 a	0.27 ± 0.0 a
Traditional		0.377 ± 0.006 b	0.347 ± 0.006 b
Cavitation		43.007 ± 1.41 b	57.003 ± 1.828 b

Traditional	Consistency Index (K)	20.177 ± 0.899 a	30.41 ± 0.57 a
Cavitation	Yield Stress	40.767 ± 0.32 b	54.093 ± 1.507 b
Traditional		15.23 ± 0.265 a	22.143 ± 0.53 a
Cavitation	R^2	0.947 ± 0.015 a	0.957 ± 0.015 ab
Traditional		0.973 ± 0.006 b	0.963 ± 0.012 ab
Cavitation	FRAP (µmol TE/100 g)	2580.409 ± 125.712 b	220.368 ± 64.782 a
Traditional		993.575 ± 109.936 a	449.122 ± 56.575 b
Cavitation	DPPH % Inhibition	6.762 ± 0.615 a	6.557 ± 0.82 b
Traditional		9.153 ± 1.317 b	3.006 ± 0.853 a
Cavitation	TPC (mg GAE/g)	2.863 ± 0.452 b	1.517 ± 0.167 b
Traditional		0.875 ± 0.033 a	0.933 ± 0.066 a
Cavitation	ORAC (µmol TE/g)	77.7 ± 7.692 b	89.867 ± 4.772 b
Traditional		44.7 ± 5.751 a	78.033 ± 5.163 a
Cavitation	Beta-Carotene (mg/L)	1.325 ± 0.036 b	0.0 ± 0.0 ab
Traditional		1.129 ± 0.022 a	0.205 ± 0.308 ab

Table S3. MRM conditions for the semi-quantification of phenolic compounds and flavonoids in positive and negative polarity by LC-QqQ.

**Negative Polarity**		
**Name**	**RT (min)**	**Transition**
Gallic acid	3.075	169.0 -> 125.0
3.5-Dihydroxybenzoic acid	4.810	152.9 -> 108.9
2.3.4-Trihydroxybenzoic acid	4.945	168.9 -> 150.9
Catechol	5.620	109.0 -> 108.0
4-Hydroxybenzoic acid	6.230	137.0 -> 93.0
Gentisic acid	6.293	153.0 -> 108.0
Chlorogenic acid	6.551	353.0 -> 191.0
(+)-Catechin (Hydrate)	6.690	289.0 -> 245.0
Dihydrocaffeic acid	6.812	180.9 -> 136.9
4-Acetocatechol	6.905	151.0 -> 108.0
Terephthalic acid	6.980	165.0 -> 121.0
Vanillic acid	7.023	167.1 -> 151.9
Resorcinol	7.160	109.0 -> 65.0
Caffeic acid	7.200	179.0 -> 135.0
Dihidroxibenzoic acids (2.3- .2.4-. 2.6-)	7.227	153.0 -> 109.0

**Negative Polarity**		
**Name**	**RT (min)**	**Transition**
3-Hydroxybenzoic acid	7.320	137.0 -> 93.0
Syringic acid	7.379	197.0 -> 182.0
Mangiferin	7.443	420.9 -> 301.0
Puerarin	7.444	415.0 -> 267.0
(-)-Epicatechin	7.710	289.0 -> 245.0
(-)-Epigallocatechin gallate hydrate	7.808	457.0 -> 169.1
4-Methylcatechol	8.600	123.0 -> 108.0
p-Coumaric	8.607	163.0 -> 119.0
Hydroferulic acid	8.843	195.0 -> 136.0
m-Hydrocoumaric acid	9.050	165.0 -> 121.0
Polydatin	9.233	389.0 -> 227.0
Rutin	9.240	609.0 -> 300.1
Ferulic acid	9.250	193.0 -> 134.0
Sinapic acid	9.289	223.0 -> 193.0
(+)-Taxifolin	9.682	303.0 -> 285.0
m-Coumaric acid	9.700	163.0 -> 119.0
Acetylphloroglucinol	9.905	167.0 -> 123.0
Naringin	10.327	579.0 -> 271.0
4-Acetylresorcinol	10.360	151.0 -> 91.0
Diosmin	10.448	607.0 -> 299.1
Hesperidin	10.613	609.0 -> 301.0
trans-2-Hydroxycinnamic acid	10.650	163.0 -> 119.0
Salicylic acid	10.727	137.0 -> 93.0
Neohesperidin	10.894	609.1 -> 286.1
Rosmarinic acid	10.980	359.0 -> 161.0
Myricetin	11.064	316.9 -> 151.0
Fisetin	11.069	285.0 -> 135.0
5-Methoxysalicylic acid	11.100	167.0 -> 107.9
Phloridzin	11.223	435.0 -> 273.0
Naringin dihydrochalcone	11.587	581.0 -> 273.0
Baicalin	11.669	444.9 -> 269.0
Resveratrol	11.727	226.9 -> 185.0
2-Acetylresorcinol	11.945	151.0 -> 91.0
Daidzein	12.091	252.9 -> 224.0
Neohesperidin DHC	12.153	611.1 -> 303.0
3.4.5-Trimethoxycinnamic acid	12.580	237.0 -> 103.0
Luteolin	12.736	284.9 -> 133.0
Quercetin	12.810	300.9 -> 121.0
Morin	12.830	301.0 -> 121.0
Apigenin	13.830	268.9 -> 151.0
(+/-)-Naringenin	13.830	270.9 -> 150.9
Genistein	13.878	268.9 -> 133.1
Phloretin	13.890	273.0 -> 167.1
Kaempferol	14.078	284.9 -> 93.1
Diosmetin	14.116	299.0 -> 284.1

**Negative Polarity**		
**Name**	**RT (min)**	**Transition**
Hesperetin	14.154	301.0 -> 163.9
Baicalein	14.400	269.0 -> 137.0
Isoliquiritigenin	14.690	255.0 -> 119.0
Formononetin	14.890	267.0 -> 252.0
Nordihydroguaiaretic Acid	15.374	301.0 -> 122.0
Chrysin	15.831	252.9 -> 63.1
Caffeic acid phenethyl ester	16.192	283.0 -> 135.0
Biochanin A	16.233	282.9 -> 268.0
α-Methyl-cinnamic acid	8.467	163.0 -> 131.0
Sinapyl Alcohol	8.550	193.0 -> 161.0
2.6-Dimethoxybenzoic acid	9.020	183.0 -> 165.0
o-Anisic Acid	9.320	153.0 -> 135.0
Veratric acid	9.530	183.0 -> 139.0
p-Anisic acid	11.017	153.0 -> 109.0
Caffeic acid dimethyl ether	11.704	209.0 -> 191.0
4-Methylsyringol	12.171	169.0 -> 137.0
3-Methoxyhydrocinnamic acid	12.965	180.9 -> 135.0
trans-Cinnamic acid	13.094	149.0 -> 131.0
4'-Hydroxychalcone	15.824	225.0 -> 207.0
Flavone	16.210	223.0 -> 121.0
trans-Chalcone	17.890	209.0 -> 103.0
